# A Comparison of the Impact of Pharmacological Treatments on Cardioversion, Rate Control, and Mortality in Data-Driven Atrial Fibrillation Phenotypes in Critical Care

**DOI:** 10.3390/bioengineering11030199

**Published:** 2024-02-20

**Authors:** Alexander Lacki, Antonio Martinez-Millana

**Affiliations:** Instituto Universitario de Investigación de Aplicaciones de las Tecnologías de la Información y de las Comunicaciones Avanzadas (ITACA), Universitat Politècnica de València, Camino de Vera S/N, 46022 Valencia, Spain; anmarmil@itaca.upv.es

**Keywords:** precision medicine, intensive care, atrial fibrillation, treatment effects, clustering

## Abstract

Critical care physicians are commonly faced with patients exhibiting atrial fibrillation (AF), a cardiac arrhythmia with multifaceted origins. Recent investigations shed light on the heterogeneity among AF patients by uncovering unique AF phenotypes, characterized by differing treatment strategies and clinical outcomes. In this retrospective study encompassing 9401 AF patients in an intensive care cohort, we sought to identify differences in average treatment effects (ATEs) across different patient groups. We extract data from the MIMIC-III database, use hierarchical agglomerative clustering to identify patients’ phenotypes, and assign them to treatment groups based on their initial drug administration during AF episodes. The treatment options examined included beta blockers (BBs), potassium channel blockers (PCBs), calcium channel blockers (CCBs), and magnesium sulfate (MgS). Utilizing multiple imputation and inverse probability of treatment weighting, we estimate ATEs related to rhythm control, rate control, and mortality, approximated as hourly and daily rates (%/h, %/d). Our analysis unveiled four distinctive AF phenotypes: (1) postoperative hypertensive, (2) non-cardiovascular mutlimorbid, (3) cardiovascular multimorbid, and (4) valvulopathy atrial dilation. PCBs showed the highest cardioversion rates across phenotypes, ranging from 11.6%/h (9.35–13.3) to 7.69%/h (5.80–9.22). While CCBs demonstrated the highest effectiveness in controlling ventricular rates within the overall patient cohort, PCBs and MgS outperformed them in specific phenotypes. PCBs exhibited the most favorable mortality outcomes overall, except for the non-cardiovascular multimorbid cluster, where BBs displayed a lower mortality rate of 1.33%/d [1.04–1.93] compared to PCBs’ 1.68%/d [1.10–2.24]. The results of this study underscore the significant diversity in ATEs among individuals with AF and suggest that phenotype-based classification could be a valuable tool for physicians, providing personalized insights to inform clinical decision making.

## 1. Introduction

Atrial fibrillation (AF) is the most prevalent cardiac arrhythmia, affecting more than 33 million patients worldwide [1]. It is commonly encountered in critically ill patients, with incidences ranging from 4.5% to 15% in intensive care units (ICUs) [2], where it is associated with higher healthcare costs, prolonged hospitalization duration, increased risk of thromboembolism, and increased mortality [3,4].

AF is a heterogeneous disease with diverse causes and mechanisms. It may be driven by cardiac and non-cardiac comorbidities, such as pulmonary, metabolic, and endocrine disorders, genetic factors, or inflammatory states [5,6]. The abundance of pathophysiological processes driving AF has led to the realization that AF is a complex arrhythmia with significant inter-patient heterogeneity [7]. To address this heterogeneity, data-driven methods such as cluster analysis have been applied to AF cohorts, identifying clinically relevant AF phenotypes with different treatment patterns and outcomes.

Hemodynamic compromise resulting from AF often makes an urgent conversion to sinus rhythm necessary in critically ill patients. In hemodynamically stable patients, AF is often observed until it terminates spontaneously, or the ventricular rate is controlled to avoid potential adverse effects associated with cardioverting antiarrhythmic drugs. Such adverse effects include thyroid disorders, hypotension, pulmonary fibrosis, and proarrhythmic effects, with some antiarrhythmics being associated higher mortality than others [8].

Within the ICU, the management of AF is mostly limited to pharmacological treatments [5,9]. Beta blockers, magnesium sulphate, and calcium channel blockers are primarily aimed at reducing the ventricular rate, by means of reducing atrioventricular node conduction. Potassium channel blockers are used to restore and maintain sinus rhythm by prolonging atrial refractory periods, thereby preventing re-entrant activity. For a comprehensive overview of the mechanisms of AF, and the molecular mechanisms of antiarrhythmic drugs, we refer the reader to [10].

The wide spectrum of treatment options coupled with a heterogeneous patient population makes treatment selection a complex endeavor. As a result, strong evidence for the optimal treatment strategy is missing [9], and AF treatment in ICUs varies across clinical institutions. Nonetheless, treatment strategies with antiarrhythmic drugs have been shown to impact patient outcomes in the short as well as the long term [11,12]. A recent multi-center survey on treatment preferences among physicians revealed a lack of consensus on whether to choose a rate control or a rhythm control strategy, a lack of consensus in the choice of antiarrhythmic agent, and a disregard for patients’ underlying pathophysiological presentation in treatment selection in 75% of respondents [13]. Even though some tendencies for treatment selection exist, they are often derived from outpatient guidelines, and are not directly applicable to ICU populations due to different AF mechanisms, risks, and effectiveness of treatments [14,15].

Previous studies have employed cluster analysis to identify and characterize different AF phenotypes in community cohorts. The first such application was performed by Inohara et al. [16], who identified four recognizable phenotypes based on 60 clinical variables. The authors observed significant differences in the use of pharmacological treatments, and rates of major adverse cardiovascular or neurological events, new-onset heart failure, hospitalization, major bleeding, and mortality. Further studies [17,18,19,20,21] incorporated different clinical variables and identified varying numbers of clusters, reporting inter-cluster differences in clinical outcomes. A recurring conclusion of previous works was that cluster analysis was able to identify clinically meaningful phenotypes, which may potentially guide treatment decisions and improve patient outcomes.

We further explore the applicability of cluster analysis and phenotype classification in AF management by assessing its ability to identify clusters with varying average treatment effects (ATEs). Hierarchical agglomerative clustering is employed to identify distinct AF phenotypes in an intensive care cohort. Phenotypes’ properties are described, and the efficacy of pharmacological interventions is evaluated, demonstrating differences in ATEs on rhythm control, rate control, as well as in-hospital mortality.

## 2. Materials and Methods

### 2.1. Data

This study performs a retrospective analysis of a large single-center intensive care database, the Medical Information Mart for Intensive Care (MIMIC-III) [22]. The MIMIC-III database contains electronic health records from 55,423 distinct ICU admissions of 46,520 patients in the critical care units of the Beth Israel Deaconess Medical Center in the years from 2001 to 2012. The data include vital signs, medications, laboratory measurements, periodically charted observations, medical procedures, diagnoses, and free-text clinical notes.

### 2.2. Cohort Definition

We include patients at least 18 years of age with a diagnostic code indicating AF (ICD-9-CM: 427.31). For patients who exhibited more than one ICU admission, only the first admission with an AF diagnosis is considered. Patients with an ICU stay shorter than 24 h or age below 18 years are excluded from the analysis.

### 2.3. Variables

Patients are described using 34 clinical variables which include comorbidities, laboratory measurements, observations, and medical history. The clinical variables were gathered based on a systematic literature review, in which clinical variables predictive of patient outcomes were identified. The employed database was screened for the presence of the variables identified in the systematic review, resulting in the 34 clinical variables presented in Table A1. Within the scope of this study, we use the earliest available record for each variable, if more than one value is available. Continuous variables are transformed into z-scores for analysis.

### 2.4. Outcomes

The primary outcomes are (i) conversion to sinus rhythm, and (ii) achievement of rate control, defined as a heart rate < 100 beats per minute [23]. In MIMIC-III patients, heart rates and heart rhythms were recorded by nurses at regular intervals, and have previously been shown to be accurate and precise to within 1 h [24]. It is assumed that a registered rhythm is maintained until a different rhythm is recorded. The secondary outcome is in-hospital mortality. The primary outcomes are censored at 24 h, and the secondary outcome is censored at 30 days [11]. For all outcomes, we consider the time from the first treatment administration until the corresponding outcome is observed.

### 2.5. Treatment Groups

The MIMIC-III database is transformed into the Observational Medical Outcomes Partnership (OMOP) Common Data Model (CDM) [25] using the code provided by [26] to identify patient exposures to different pharmaceutical agents. Treatments are captured based on the ingredients in administered drugs, which are accompanied by the timestamp indicating when the treatment was initiated. Within the scope of this study, four treatment groups are defined based on classes 2–4 of the Vaughan Williams classification [27]—beta blockers (BBs), potassium channel blockers (PCBs), and calcium channel blockers (CCBs), as well as magnesium sulphate (MgS). The treatment groups with the corresponding drug ingredients are shown in Table 1. For the outcomes conversion to sinus rhythm and in-hospital mortality, treatment group assignment is determined based on the first observed drug exposure during an AF episode, while for the rate control, it is determined based on the first observed drug exposure during an AF episode with a rapid ventricular response (>100 beats per minute).

### 2.6. Multiple Imputation

We handled missing data through multiple imputation [28]. To achieve this, linear regression models were utilized to generate imputed datasets. This process involved resampling the original dataset with replacement, leading to the creation of 60 bootstrapped datasets. To fit linear regression models to these bootstrapped datasets, we employed chained equations [29], a technique that effectively accounts for the interrelationships among descriptive variables. Subsequently, the original dataset underwent repeated imputation using these models, resulting in a total of 60 imputed datasets. With a fraction of missing information amounting to 7.42%, the number of imputed datasets adheres to Bodner’s rule [30].

### 2.7. Inverse Probability of Treatment Weighting

Given the nature of our retrospective cohort analysis, it is important to consider that patients may have received treatments in a non-randomized manner, based on their specific pathophysiological presentations. Failing to account for this selection bias during the evaluation of treatment effects could lead to biased treatment effects [31].

To address the influence of confounding variables, we utilize a statistical method called inverse probability of treatment weighting (IPTW), implemented through the Twang toolkit [32]. Within each imputed dataset, we compute the likelihood of patients being allocated to their specific treatment categories based on their descriptive attributes, employing gradient boosted logistic regression models. This methodology allows us to quantify the likelihood of patients receiving a specific treatment based on their individual characteristics. The inverse of this likelihood score for each patient serves as a weighting factor in subsequent analyses to mitigate the impact of confounding variables.

We evaluate the balance of covariates across treatment groups by computing the maximum absolute pairwise standardized mean differences. This measure allowed us to assess the degree of covariate imbalance between the treatment groups, with smaller differences indicating improved balance.

The hyperparameters employed for the IPTW method were determined empirically, taking into consideration the total computation time and obtained covariate balance.

### 2.8. Cluster Analysis

Patient phenotypes are identified using hierarchical agglomerative clustering. We use a complete linkage criterion for agglomeration, and Gower’s distance metric [33] to account for the combination of continuous and categorical covariates. Pairwise distances are computed for each imputed dataset, and averaged to a single distance matrix, which is used in the clustering algorithm. The number of clusters is defined in a compromise between resolution and clinical explainability. The number of clusters is manually chosen such that recognizable patient groups are apparent, blinded to treatments and outcomes. Patient characteristics are compared among the different clusters, and statistical differences are assessed using the Kruskal–Wallis test. Patient covariates are described in terms of their medians and interquartile ranges (IQRs) for continuous covariates, while categorical covariates are reported as counts and percentages. Each cluster is described in terms of its most prominent properties to provide an intuitive characterization.

### 2.9. Statistical Analysis

The key component of the statistical analysis is the estimation of ATEs. Different approaches may be taken to analyze and present such data, such as univariate and multivariate Cox analyses and multiparametric and exponential survival models [34]. To provide the highest degree of interpretability, we approximate ATEs using weighted exponential survival models with weights obtained from IPTW.

The survival models are fitted to each imputed dataset 100 times, utilizing Bayesian bootstrapping as proposed by Rubin [35]. This process results in a total of 6000 event rate estimates for each ATE. The estimates of constant event rates are subsequently presented as probability distributions. We report both the mode and the 95% highest density intervals of these distributions.

ATEs are computed for the complete cohort and for individual clusters. This allows us to examine the effects of the treatment both overall and within specific clusters. We assess differences in ATEs using Bayes factors (BFs) [36] to provide a quantifiable uncertainty estimate in the effects of different treatments.

Our results are reported as hourly rates (%/h) for the primary outcomes, and as daily rates (%/d) for the secondary outcome. This allows for a clear and direct comparison of the effects of different treatments over time.

All statistical analyses were performed using Python 3.7 and R Core v4.1.2. A secondary analysis using the KMeans algorithm can be found in Section S1 of the Supplementary Material.

## 3. Results

Of the 46,520 patients in the database, 10,277 have a diagnostic code indicating AF. After excluding patients with age below 18 years and a hospital stay shorter than 24 h, a total of 9401 patients were included in the analysis. The cohort characteristics are shown in Table 2. The percentages of missing values for each characteristic are shown in Table A1 in Appendix A. Patients were followed for an average of 11.3 days (IQR, 5.29–13.9), and were either discharged after 5.28 days (IQR, 10.2–12.0), or expired after 5.35 days (IQR, 12.4–15.9). The share of in-hospital mortality was 49.7%.

### 3.1. Patient Clusters

**Figure 1:**
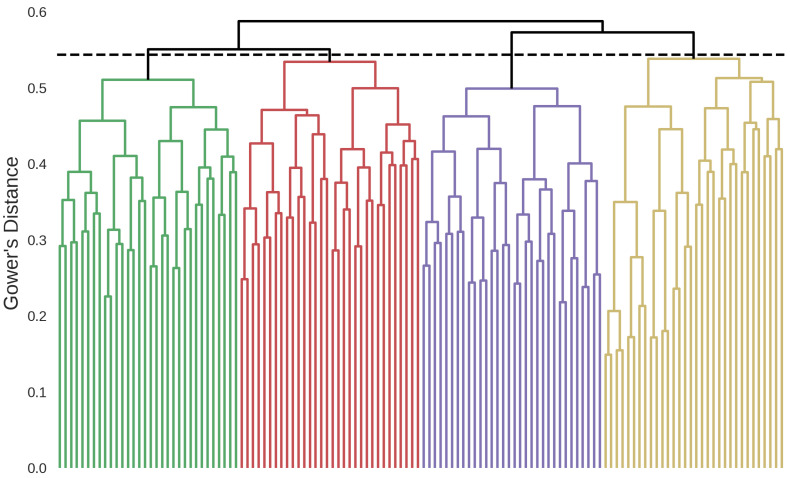
Hierarchical clustering dendrogram showing the four identified clusters. Green: Postoperative Hypertensive, red: Non-Cardiovascular Multimorbid, purple: Cardiovascular Multimorbid, yellow: Valvulopathy Atrial Dilation. The dashed horizontal line represents the stopping criterion used for the final cluster definition.

A total of four clusters were identified based on the hierarchical clustering dendrogram presented in Figure 1. The clinical variables of the identified phenotypes are shown in Table A2 in Appendix A. The dominant characteristics of the identified patient clusters are as follows.

**Cluster 1: Postoperative Hypertensive (n = 2963)**
  Patients in this cluster have the highest rate of postoperative conditions (74.2%) and hypertension (64.3%). This cluster has the highest share of male patients (72.1%), and the highest rate of coronary artery atherosclerosis (65.5%). Notably, patients in this cluster are the youngest (median age, 73.3; IQR, 65.1–80.5), have the lowest heart rates (median, 83.5; IQR, 75.0–90.1), and the lowest rate of arrhythmia history (12.8%).

**Cluster 2: Non-Cardiovascular Multimorbid (n = 3546)**
  This cluster is characterized by the highest rate of chronic obstructive pulmonary disease (COPD) (16.6%) and diabetes (25.8%), while also having a high rate of renal insufficiency (43.9%). Patients in this cluster have the lowest rate of left and right atrial dilation (17.9%, 6.49%), coronary artery atherosclerosis (18.3%), and valvulopathies (12.6%) while having the highest rate of arrhythmia history (26.3%).

**Cluster 3: Cardiovascular Multimorbid (n = 557)**
  Patients in this cluster are the oldest (median age, 78.1; IQR, 69.2–85.4) and have the highest rate of heart failure (85.5%). They have the highest rate of left and right atrial dilation (94.3%, 78.8%), thyroid disorders (16.0%), myocardial infarction (19.0%), renal insufficiency (54.6%), respiratory failure (40.2%), sepsis (30.9%), and obstructive sleep apnea (7.36%). Even though patients in this cluster have a high rate of comorbidities, they have the lowest rate of hypertension (27.1%).

**Cluster 4: Valvulopathy Atrial Dilation (n = 2335)**
  This cluster is characterized by the highest rate of valvulopathies (45.2%) and a high rate of left and right atrial dilation (91.0%, 76.0%). Patients in this cluster further have the highest rates of cor pulmonale (14.3%) and a high rate of COPD (16.0%).

### 3.2. Treatment Effects

IPTW resulted in well-matched covariates between treatment groups within all outcomes. Variable means and maximum absolute pairwise standardized mean differences for treatment groups are shown in Supplementary Tables S2 and S3. The complete list of treatment effects is available in Supplementary Tables S4–S6.

#### 3.2.1. Rhythm Control

**Figure 2:**
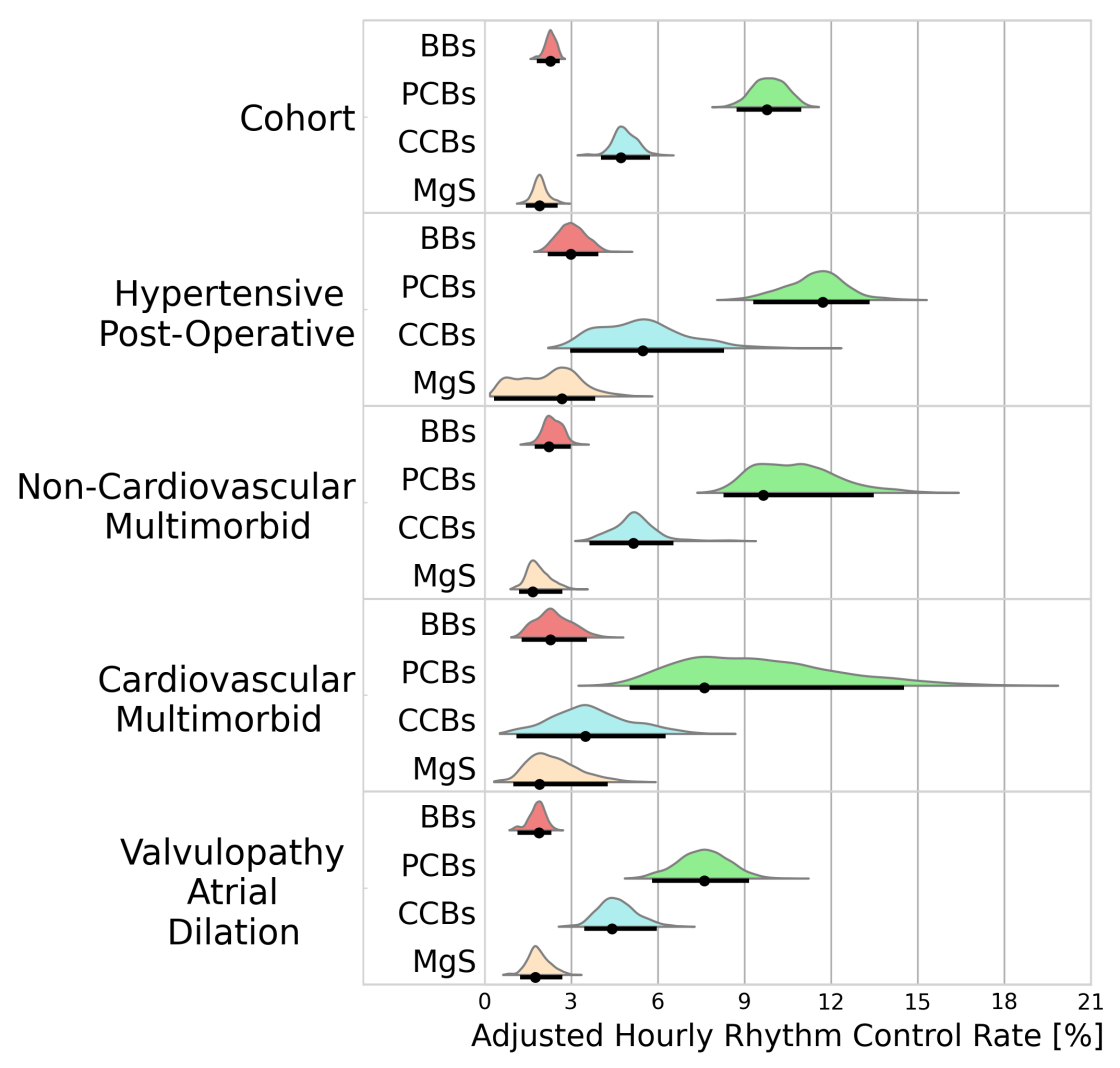
Probability distributions of adjusted hourly rhythm control rates. Black dots represent the distribution modes. Black lines represent the 95% highest density intervals. BBs—beta blockers; PCBs—potassium channel blockers; CCBs—calcium channel blockers; MgS—magnesium sulphate.

A total of 4116 patients received a treatment during an AF episode. BBs, PCBs, CCBs, and MgS were administered to 1277, 1388, 830, and 502 patients, respectively. Figure 2 portrays the adjusted hourly rhythm control rates for the cohort analysis and the individual clusters. Rhythm control was best achieved using PCBs (9.78%/h [8.74–11.0]), followed by CCBs (4.73%/h [4.04–5.74]). Inferior rhythm control was observed in patients receiving BBs (2.28%/h [1.81–2.61]) and MgS (1.90%/h [1.44–2.54]).

Within the identified clusters, PCBs maintained superiority, but its efficacy varied considerably with higher conversion rate in patients in the hypertensive postoperative cluster than in patients in the valvulopathy atrial dilation cluster (11.6%/h [9.35–13.3] vs. 7.69%/h [5.80–9.22], BF > 100).

#### 3.2.2. Rate Control

**Figure 3:**
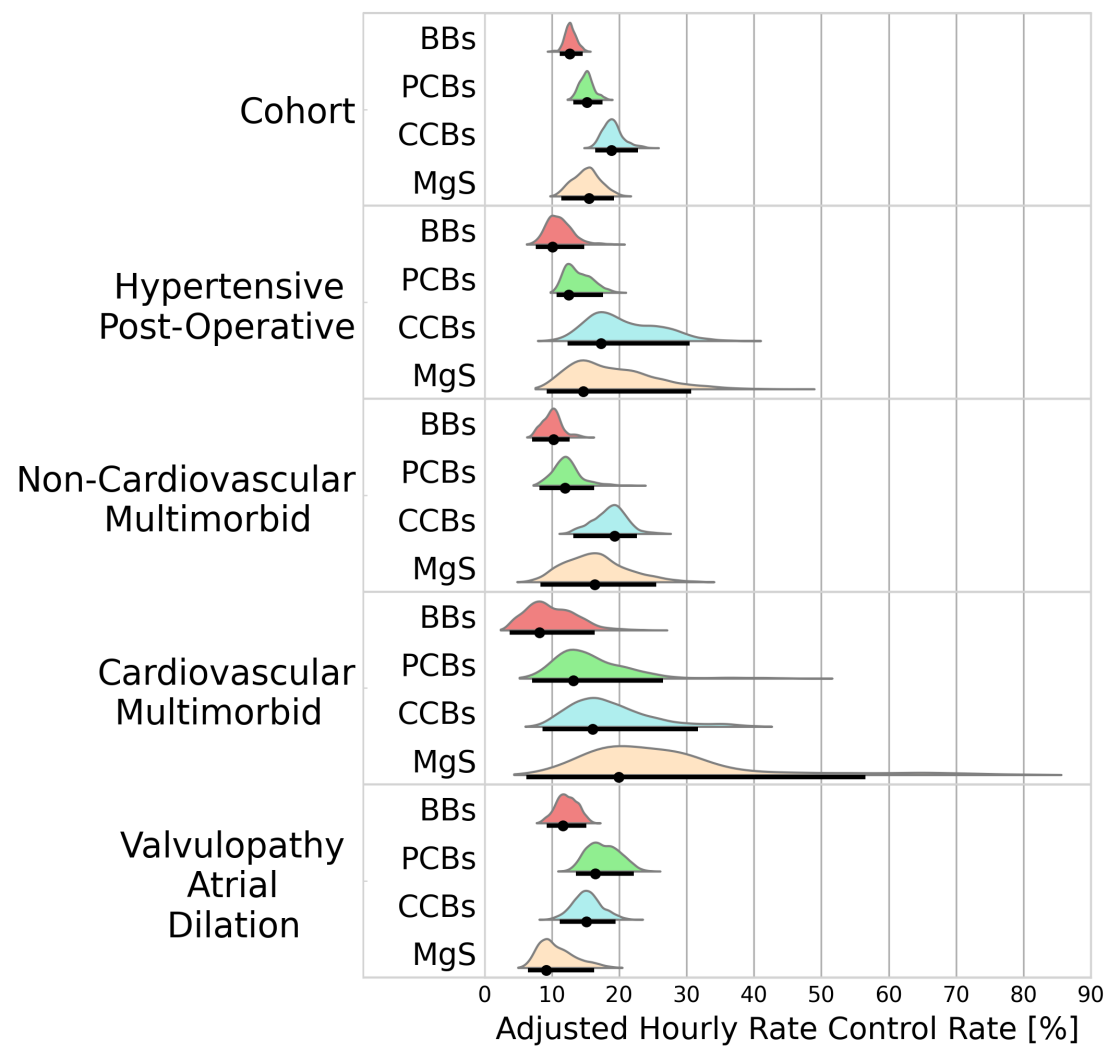
Probability distributions of adjusted hourly rate control rates. Black dots represent the distribution modes. Black lines represent the 95% highest density intervals. BBs—beta blockers; PCBs—potassium channel blockers; CCBs—calcium channel blockers; MgS—magnesium sulphate.

A total of 2333 patients received a treatment during an AF episode with rapid ventricular response. BBs, PCBs, CCBs, and MgS were administered to 566, 966, 644, and 157 patients, respectively. Rate control was best achieved using CCBs (18.8%/h [16.4–22.8]) followed by MgS (15.6%/h [11.4–19.2). Inferior rate control was observed for patients receiving PCBs and BBs (15.2%/h [13.2–17.5], 15.6%/h [11.4–19.2]). Figure 3 portrays the adjusted hourly rates for HR reduction below 100 bpm.

The superiority of CCBs observed in the cohort analysis was not maintained among all clusters. Even though CCBs showed a higher efficacy at controlling ventricular rate than MgS in the entire cohort (BF = 14.8), the opposite was observed in the cardiovascular multimorbid cluster (18.3%/h [11.2–32.6] vs. 19.9%/h [7.92–57.5], BF = 0.40). Similarly, while CCBs outperformed PCBs in the cohort analysis (BF = 68.6), PCBs appeared to have a higher efficacy than CCBs in the valvulopathy atrial dilation cluster (18.2%/h [14.9–23.6] vs. 16.7%/h [12.6–20.9], BF = 3.26). While MgS showed superiority to BBs in the entire cohort analysis (BF = 5.83), the opposite was observed in the valvulopathy atrial dilation cluster (BF = 0.42).

#### 3.2.3. Mortality

**Figure 4:**
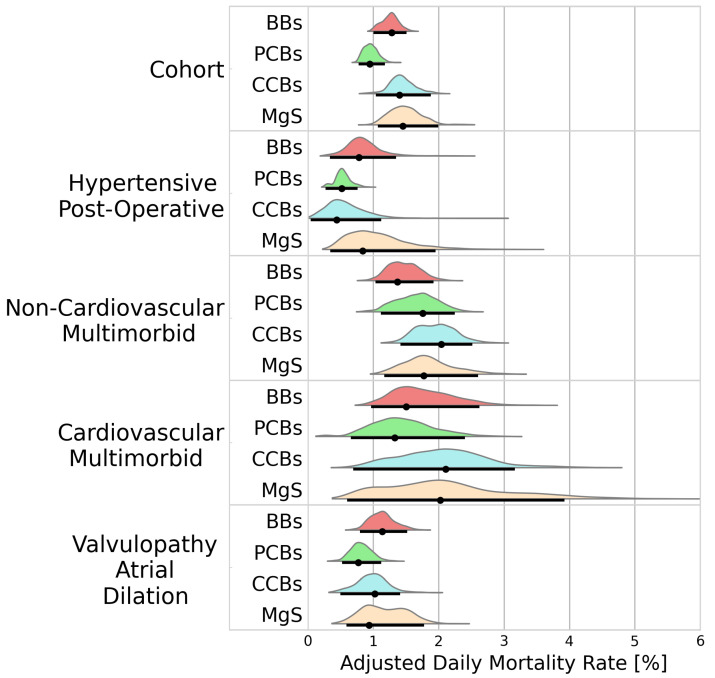
Probability distributions of adjusted daily mortality rates. Black dots represent the distribution modes. Black lines represent the 95% highest density intervals. BBs—beta blockers; PCBs—potassium channel blockers; CCBs—calcium channel blockers; MgS—magnesium sulphate.

In-hospital mortality differed across treatment groups, with the highest mortality being observed in patients receiving MgS (1.45%/d [1.07–1.99]), followed by CCBs and BBs (1.40%/d [1.04–1.88], 1.28%/d [1.00–1.51]). The lowest mortality was observed for PCBs (0.95%/d [0.78–1.18]). Figure 4 presents the adjusted daily mortality rates for the cohort analysis, and the individual clusters.

The mortality rates varied across the identified clusters. While PCBs were associated with the lowest mortality rates in the cohort analysis, this did not hold true within the hypertensive post-operative cluster, where mortality rates with PCBs were comparable to CCBs (0.52%/d [0.28–0.76] vs. 0.44%/d [0.05–1.15], BF = 1.02). In the non-cardiovascular multimorbid cluster, PCBs were associated with a higher mortality rate than BBs (1.68%/d [1.10–2.24] vs. 1.33%/d [1.04–1.93], BF = 2.23).

The results of the secondary analysis can be found in the supplementary material, and include: The cluster characteristics of the secondary analysis (Table S1), and probability distributions of adjusted treatment effects for the obtained clusters (Figures S1–S3).

## 4. Discussion

This study investigated clustering-derived AF phenotypes and their treatment effects in an ICU cohort. The main findings of the study are as follows. (i) The heterogeneity in the AF population, previously reported in community cohorts, can also be observed in the analyzed ICU population. (ii) ATEs differ between phenotypes and are often different from those observed when the treatment effect is averaged across the entire population.

In accordance with previous cluster analyses of AF populations, the analysis of the ICU cohort revealed recognizable patient groups, for example, the postoperative hypertensive cluster, which was characterized by young age and predominantly male patients. With postoperative conditions being known for triggering AF by means of causing inflammatory states, patients in this cluster had the lowest rates of arrhythmia history, and, thus, the highest proportion of new-onset AF.

Previous studies have utilized cluster analysis to explore the differences in treatment patterns and clinical outcomes in AF cohorts. Our work expands on this research by demonstrating that AF phenotypes derived from cluster analysis also exhibit heterogeneity in terms of ATEs. This finding is consistent with previous studies that have criticized the reporting of overall mean effects in clinical research, as such an approach may overlook important treatment effects that are specific to certain patient subgroups [37]. The observed heterogeneity in ATEs in this study carries major significance for clinical research. ATEs are commonly derived from the analysis of entire cohorts and heterogeneous treatment effects go unnoticed. For example, a previous study reported benefits in mortality of BBs versus CCBs [38], which we can confirm when analyzing the ATE for the complete cohort, but we find evidence of the opposite effect within two of the identified clusters. Similarly, we can confirm previous results showing that CCBs outperform PCBs in controlling ventricular rate [39] when considering the ATE on the entire cohort. Evaluating individual phenotypes, however, reveals evidence for the opposite effect for patients with valvulopathies and dilated atria. The identification of AF phenotypes may provide insight for further study design, and provide a method to evaluate heterogeneous cohorts such that heterogeneous treatment effects are not overlooked.

While heterogeneities in ATEs were observed among the identified phenotypes, several relationships did not appear as would be expected. Several contraindications of the investigated treatments are known, such as CCBs being contraindicated in patients with systolic heart failure, and amiodarone (PCB) in patients with thyroid disorders. Ideally, one would expect clusters characterized by such contraindications to emerge, and the expected ATEs to be reflected in the results. To what extent such clusters may emerge when a more fine-grained clustering is performed must be evaluated. Given our analysis does not reveal these existing treatment effects, it must be understood that we do not propose a formal classification.

Nonetheless, the presented results may have major clinical implications. A recent survey has shown that 75% of clinical decision-makers treating AF in the ICU would not change their intervention strategy depending on an underlying pathophysiological condition [13]. Our results underline the necessity of considering the pathophysiological presentation of patients during treatment selection, to maximize treatment utility and to minimize risk. Overlooking the heterogeneity in AF patients may result in inadequate treatment and lead to suboptimal patient outcomes.

Numerous studies [16,18,19,20,21] have suggested that a phenotype-driven approach for the management of AF may improve patient outcomes by either providing insights that drive further research, or by guiding treatment directly. However, some studies have reported conflicting results, such as phenotypes with high rates of anticoagulation being associated with increased incidence of ischemic events [16,18]. We have therefore provided ATEs in this work to further expand the idea of a phenotype classification for AF management. Such an approach can help quantify treatment effects for specific patient phenotypes, which can aid in selecting appropriate treatments to maximize desired effects while minimizing the probability of adverse outcomes. However, in order to implement such a quantification approach, additional studies are needed to validate our findings in prospective multi-center cohorts and determine the extent to which the results can be generalized beyond the specific cohort used in our study.

### Limitations

The results of this study should be interpreted within the context of several limitations. First, the database used does not provide adequate temporal resolution for procedural and diagnostic codes, which are generated when a patient is discharged. It must therefore be understood that the obtained results inevitably suffer from look-ahead bias. Second, the selection of descriptive variables has profound impact on the results of cluster analysis. While care was taken to select relevant variables, candidate variables had to be removed due to data sparsity or were completely unavailable in the database. The inclusion of further variables may impact the results and reduce residual confounding. Third, the present study defined treatment groups according to the first treatment received during an AF episode. In practice, treatments may be administered in combinations, or incrementally escalated until the desired effect is achieved. Further, we have only considered a limited number of treatments due to relatively infrequent use of alternative antiarrhythmic drugs. A more extensive dataset may provide insights into the treatment effects of further antiarrhythmic agents. Fourth, while the employed unsupervised clustering algorithm has previously been shown to identify clinically relevant patient phenotypes, our approach of deciding the number of clusters was primarily based on investigator discretion. A common practice in unsupervised clustering would be the identification of the optimal number of clusters using a clustering metric, such as the silhouette score or the Calinski–Harabasz index. Such approaches have, however, not shown any usable results in our dataset, indicating a lack of structure in the covariate space. Other studies [40,41] have proposed semi-supervised cluster analysis to determine the appropriate number of clusters. Such methods form clusters that correlate with outcomes, identifying patient phenotypes with increased clinical significance. Finally, while the obtained results show significant differences in treatment effects, the obtained distributions were often too wide to give conclusive results. The use of a larger dataset may allow for more discriminatory results.

## 5. Conclusions

Cluster analysis of the employed ICU cohort identified four recognizable AF phenotypes defined by unique characteristics. Phenotypes showed different treatment effects, highlighting the heterogeneity of AF patients in critical care settings. Our results support the idea of a phenotype classification approach to support clinical decision making by quantifying treatment effects of individual patients and provide a basis for the design of further studies. Future works should consider the application of semi-supervised clustering methods to emphasize cohesive treatment effects in the formation of clusters, thereby maximizing their clinical significance.

## Figures and Tables

**Table 1:** Treatment groups and their corresponding drug ingredients.

Treatment Group	Active Principles	Mechanism of Action
Beta Blockers	Acebutolol	Decrease sympathetic activity by blocking ß-adrenergic receptors, reducing AV node conduction and calcium influx.
	Esmolol	
	Labetalol	
	Metoprolol	
	Propranolol	
Potassium Channel Blockers	Amiodarone	Prolong the duration of action potentials and refractory periods, preventing re-entrant activity.
	Dofetilide	
	Dronedarone	
	Ibutilide	
	Sotalol	
Calcium Channel Blockers	Dilatiazem Verapamil	Decrease conduction through the AV node, and shorten phase two of the cardiac action potential, decreasing myocardial contraction.
Magnesium Sulphate	Magnesium Sulphate	Stabilizes the membrane potential, prolongs refractory periods, and decreases AV node conduction and sinus node recovery times.

**Table 2:** Cohort characteristics.

Category	Characteristic	Median (IQR)/n (%)
Medical History	Anemia	1127 (11.99)
	Arrhythmia History	1959 (20.8)
	COPD	1256 (13.4)
	Collagen disease	115 (1.22)
	Cor pulmonale	754 (8.02)
	Coronary artery atherosclerosis	3461 (36.8)
	Diabetes	2147 (22.8)
	Thyroid disorder	1051 (11.2)
	Heart failure	4128 (43.9)
	Hypertension	4562 (48.5)
	Myocardial infarction	933 (9.92)
	OSA	403 (4.29)
Medical History	Post-operative condition	3250 (34.6)
	Renal insufficiency	3261 (34.7)
	Respiratory failure	1450 (15.4)
	Rheumatism	403 (4.29)
	Sepsis	1144 (12.2)
	Valvulopathy	2865 (30.5)
Laboratory Measurements	Erythrocyte distribution width [ratio]	14.4 (13.5–15.7)
	Erythrocyte count [#/µm]	3.88 (3.38–4.37)
	Serum calcium [mg/dL]	8.60 (8.10–9.10)
	Serum creatinine [mg/dL]	1.10 (0.80–1.50)
	Serum magnesium [mg/dL]	2.00 (1.80–2.20)
	Serum potassium [mmol/L]	4.20 (3.90–4.70)
	Serum sodium [mmol/L]	139 (136–141)
	Hemoglobin [g/dL]	12.1 (10.6–13.4)
	Leukocyte count [#/nL]	10.0 (7.30–13.7)
	Platelet count [#/nL]	215 (163–279)
	Prothrombin time [s]	14.5 (13.1–18.1)
Observation	Heart Rate [BPM]	85.5 (75.0–98.0)
	Left Atrial Dilation	4183 (44.5)
	Right Atrial Dilation	2813 (29.9)
Demographics	Age [years]	76.5 (67.3–83.6)
	Male sex	5364 (57.1)

## Data Availability

The data used in this study are openly available, and can be found in the PhysioNet archive [42].

## Supplementary Materials

The following supporting information can be downloaded at: https://www.mdpi.com/article/10.3390/bioengineering11030199/s1, Table S1: Characteristics of the cohort, and the identified clusters of the secondary analysis. Table S2: Covariate means before and after inverse probability treatment weighting for rhythm control and mortality. Table S3: Covariate means before and after inverse probability treatment weighting for rate control. Table S4: Average treatment effects for the entire cohort and phenotypes-rhythm control. Table S5: Average treatment effects for the entire cohort and phenotypes-rate control. Table S6: Average treatment effects for the entire cohort and phenotypes-in-hospital mortality. Figure S1: Probability distributions of adjusted hourly rhythm control rates of the secondary analysis. Figure S2: Probability distributions of adjusted hourly rate control rates of the secondary analysis. Figure S3: Probability distributions of adjusted daily mortality rates of the secondary analysis.

## Abbreviations

The following abbreviations are used in this manuscript:

AF	Atrial fibrillation
ATE	Average treatment effect
BB	Beta blocker
BF	Bayes factor
CCB	Calcium channel blocker
COPD	Chronic obstructive pulmonary disease
HDI	Highest density interval
ICU	Intensive care unit
IQR	Interquartile range
MgS	Magnesium sulphate
NOAF	New onset atrial fibrillation
OSA	Obstructive sleep apnoe
PCB	Potassium channel blocker

## Appendix A

**Table A1:** Percentage of data missing and source for each patient characteristic. Source codes are SNOMED unless stated otherwise.

Category	Characteristic	Missing (%)	Standard Name	Source Code
Medical History	Anemia	0.00	Anemia	271737000
	Arrhythmia history	0.00	Arrhythmia history	MIMIC ITEMID: 225811
	Collagen disease	0.00	Systemic lupus erythematosus	55464009
			Polymyalgia rheumatica	65323003
			Sjögren’s syndrome	83901003
			81573002	81573002
	COPD	0.00	Chronic obstructive lung disease	13645005
	Cor pulmonale	0.00	Acute cor pulmonale	49584005
			Chronic pulmonary heart disease	87837008
			Pulmonary arterial hypertension	11399002
	Coronary artery atherosclerosis	0.00	Coronary arteriosclerosis	53741008
	Diabetes	0.00	Type 1 diabetes mellitus	46635009
			Type 2 diabetes mellitus	44054006
			Diabetes insipidus	15771004
			Diabetes mellitus	73211009
			Type 2 diabetes mellitus without complication	313436004
			Nephrogenic diabetes insipidus	111395007
			Diabetic ketoacidosis	420422005
Medical History	Heart failure	0.00	Congestive rheumatic heart failure	82523003
			Acute systolic heart failure	443254009
			Diastolic heart failure	418304008
			Congestive heart failure	42343007
			Chronic diastolic heart failure	441530006
			Acute diastolic heart failure	443343001
			Heart failure	84114007
			Systolic heart failure	417996009
			Acute on chronic systolic heart failure	443253003
			Acute on chronic diastolic heart failure	443344007
			Chronic systolic heart failure	441481004
			Acute combined systolic and diastolic heart failure	153931000119109
			Acute on chronic combined systolic and diastolic heart failure	153951000119103
			Chronic combined systolic and diastolic heart failure	153941000119100
	Hypertension	0.00	59621000	
	Myocardial infarction	0.00	Acute subendocardial infarction	70422006
			Myocardial infarction	22298006
	OSA	0.00	Obstructive sleep apnea syndrome	78275009
	Post-operative condition	0.00	Percutaneous transluminal coronary angioplasty [PTCA]	OMOP:2000064
			Insertion of non-drug-eluting coronary artery stent(s)	OMOP:2001505
			Angioplasty of other non-coronary vessel(s)	OMOP:2002222
			Multiple vessel percutaneous transluminal coronary angioplasty [PTCA] or coronary atherectomy performed during the same operation, with or without mention of thrombolytic agent	OMOP:2001504
			Open and other replacement of aortic valve	OMOP:2001450
			(Aorto)coronary bypass of four or more coronary arteries	OMOP:2001513
			Open heart valvuloplasty of mitral valve without replacement	OMOP:2001444
			(Aorto)coronary bypass of two coronary arteries	OMOP:2001511
			Open and other replacement of mitral valve with tissue graft	OMOP:2001451
			Open and other replacement of mitral valve	OMOP:2001452
			Insertion of drug-eluting coronary artery stent(s)	OMOP:2001506
			Extracorporeal circulation auxiliary to open heart surgery	OMOP:2002243
			Open and other replacement of aortic valve with tissue graft	OMOP:2001449
			Pericardiotomy	OMOP:2001533
			(Aorto)coronary bypass of three coronary arteries	OMOP:2001512
			(Aorto)coronary bypass of one coronary artery	OMOP:2001510
Medical History	Renal insufficiency	0.00	Renal failure syndrome	42399005
			Chronic kidney disease	709044004
			Acute renal failure syndrome	14669001
			Chronic kidney disease stage 1	431855005
			Chronic kidney disease stage 2	431856006
			Chronic kidney disease stage 3	433144002
			Chronic kidney disease stage 4	431857002
			Chronic kidney disease stage 5	433146000
			Benign hypertensive renal disease with renal failure	698591006
			End-stage renal disease	46177005
			Chronic kidney disease stage 5 due to hypertension	129161000119100
	Respiratory failure	0.0 0	Acute respiratory failure	65710008
	Rheumatism	0.00	Rheumatic aortic stenosis	72011007
			Rheumatic mitral regurgitation	31085000
			Rheumatic aortic stenosis with insufficiency	17759006
			Congestive rheumatic heart failure	82523003
			Rheumatic disease of aortic valve	12023003
			Rheumatoid arthritis	69896004
			Rheumatic heart disease	23685000
			Rheumatic disease of pulmonary valve	18687009
			Rheumatic aortic regurgitation	78031003
	Sepsis	0.00	Sepsis	91302008
			Septic shock	76571007
			Severe sepsis	1036671000000106
	Thyroid disorder	0.00	Primary malignant neoplasm of thyroid gland	94098005
			Congenital hypothyroidism	190268003
			Thyrotoxic crisis	29028009
			Benign neoplasm of thyroid gland	92439006
			Acquired hypothyroidism	111566002
			Disorder of thyroid gland	14304000
Medical History	Valvulopathy	0.00	Mitral valve regurgitation	48724000
			Mitral and aortic incompetence	194736003
			Ebstein’s anomaly	204357006
			Subaortic stenosis	204368006
			Mitral valve stenosis	79619009
			Trauma and postoperative pulmonary insufficiency	266370006
			Rheumatic aortic stenosis	72011007
			Mitral stenosis and aortic insufficiency	194734000
			Mitral and aortic stenosis	194733006
			Rheumatic mitral regurgitation	31085000
			Rheumatic aortic stenosis with regurgitation	17759006
			Aortic valve disorder	8722008
			Aortic valve regurgitation	60234000
			Tricuspid valve disorder	20721001
			Mitral insufficiency and aortic stenosis	194735004
			Mitral valve disorder	11851006
			Aortic valve stenosis	60573004
			Rheumatic disease of aortic valve	12023003
			Rheumatic aortic regurgitation	78031003
			Mitral stenosis with insufficiency	194726006
Laboratory Measurement	Erythrocyte count [#/µm]	0.97	Erythrocytes [#/volume] in Blood by Automated count	LOINC:789-8
	Erythrocyte distribution width [ratio]	0.98	Erythrocyte distribution width [Ratio] by Automated count	LOINC:788-0
	Hemoglobin [g/dL]	0.88	Hemoglobin [Mass/volume] in Blood	LOINC:718-7
	Leukocyte count	0.96	Leukocytes [#/volume] in Blood by Manual count	LOINC:804-5
	Platelet count	0.93	Platelets [#/volume] in Blood by Manual count	LOINC:778-1
			Platelets [#/volume] in Blood by Automated count	LOINC:777-3
	Prothrombin time	1.80	Prothrombin time (PT)	LOINC:5902-2
	Serum calcium [mg/dL]	5.07	Calcium [Mass/volume] in Serum or Plasma	LOINC:17861-6
	Serum creatinine [mg/dL]	0.87	Creatinine [Mass/volume] in Serum or Plasma	LOINC:2160-0
	Serum magnesium [mg/dL]	1.40	Magnesium [Moles/volume] in Serum or Plasma	LOINC:2601-3
	Serum potassium [mmol/L]	1.03	Potassium [Moles/volume] in Serum or Plasma	LOINC:2823-3
	Serum sodium [mmol/L]	1.04	Sodium [Moles/volume] in Serum or Plasma	LOINC:2951-2
Observation	Heart Rate [BPM]	2.34	Heart rate	LOINC:8867-4
	Left Atrial Dilation	0.00	-	String matching from clinical notes
	Right Atrial Dilation	0.00	-	String matching from clinical notes
Demographic	Age	0.00	-	-
	Male sex	0.00	“MALE”/”FEMALE”	OMOP:8507 OMOP:8532

**Table A2:** Characteristics of the cohort, and the identified clusters.

Category	Variable	Entire Cohort (n = 9401)	Cluster 1 (n = 2963)	Cluster 2 (n = 3546)	Cluster 3 (n = 557)	Cluster 4 (n = 2335)	*p*-Value (among Clusters)
Medical History	Anemia	1127 (11.99)	390 (13.16)	369 (10.41)	70 (12.57)	298 (12.76)	<0.001
	Arrhythmia History	1959 (20.8)	380 (12.8)	932 (26.3)	113 (20.3)	534 (22.9)	<0.001
	Collagen disease	115 (1.22)	24 (0.81)	58 (1.64)	11 (1.97)	22 (0.94)	<0.001
	COPD	1256 (13.4)	226 (7.63)	588 (16.6)	68 (12.2)	374 (16.0)	<0.001
	Cor pulmonale	754 (8.02)	179 (6.04)	164 (4.62)	77 (13.8)	334 (14.3)	<0.001
	Coronary artery atherosclerosis	3461 (36.8)	1941 (65.5)	650 (18.3)	223 (40.0)	647 (27.7)	<0.001
	Diabetes	2147 (22.8)	677 (22.9)	915 (25.8)	101 (18.1)	454 (19.4)	<0.001
	Heart failure	4128 (43.9)	851 (28.7)	1441 (40.6)	476 (85.5)	1360 (58.2)	<0.001
	Hypertension	4562 (48.5)	1924 (64.9)	1401 (39.5)	151 (27.1)	1085 (46.5)	<0.001
	Myocardial infarction	933 (9.92)	403 (13.6)	167 (4.71)	106 (19.0)	257 (11.0)	<0.001
	OSA	403 (4.29)	130 (4.39)	126 (3.55)	41 (7.36)	106 (4.54)	<0.001
	Post-operative condition	3250 (34.6)	2198 (74.2)	215 (6.06)	92 (16.5)	745 (31.9)	<0.001
	Renal insufficiency	3261 (34.7)	519 (17.5)	1555 (43.9)	304 (54.6)	883 (37.8)	<0.001
	Respiratory failure	1450 (15.4)	118 (3.98)	819 (23.1)	224 (40.2)	289 (12.4)	<0.001
	Rheumatism	403 (4.29)	126 (4.25)	82 (2.31)	33 (5.92)	162 (6.94)	<0.001
	Sepsis	1144 (12.2)	56 (1.89)	651 (18.4)	172 (30.9)	265 (11.4)	<0.001
	Thyroid disorder	1051 (11.2)	246 (8.30)	398 (11.2)	89 (16.0)	318 (13.6)	<0.001
	Valvulopathy	2865 (30.5)	1230 (41.5)	446 (12.6)	133 (23.9)	1056 (45.2)	<0.001
Laboratory Measurement	Erythrocyte count [#/μm]	3.88 (3.38–4.37)	3.88 (3.32–4.39)	3.86 (3.39–4.35)	3.82 (3.31–4.23)	3.91 (3.44–4.39)	<0.001
	Erythrocyte distribution width [ratio]	14.4 (13.5–15.7)	13.9 (13.2–14.7)	14.7 (13.7–16.2)	15.1 (13.9–16.8)	14.5 (13.7–15.9)	<0.001
	Hemoglobin [g/dL]	12.1 (10.6–13.4)	12.6 (11.3–13.8)	11.7 (10.2–13.2)	11.5 (10.1–12.8)	12.0 (10.6–13.4)	<0.001
	Leukocyte count [#/nL]	10.0 (7.30–13.7)	9.4 (7.10–12.6)	10.6 (7.50–14.6)	10.6 (7.60–14.7)	9.90 (7.10–13.4)	<0.001
	Platelet count [#/nL]	215 (163–279)	197 (153–249)	230 (171–299)	212 (163–285)	222 (167–286)	<0.001
	Prothrombin time [s]	14.5 (13.1–18.1)	14.1 (13.0–16.0)	14.7 (13.1–20.2)	15.2 (13.4–20.7)	14.6 (13.2–19.0)	<0.001
	Serum calcium [mg/dL]	8.60 (8.10–9.10)	8.6 (8.20–9.00)	8.60 (8.10–9.00)	8.60 (8.10–9.10)	8.70 (8.20–9.10)	<0.001
	Serum creatinine [mg/dL]	1.10 (0.80–1.50)	1.00 (0.80–1.20)	1.10 (0.80–1.70)	1.30 (0.90–1.90)	1.10 (0.90–1.50)	<0.001
	Serum magnesium [mg/dL]	2.00 (1.80–2.20)	2.00 (1.80–2.30)	1.90 (1.70–2.20)	2.00 (1.80–2.30)	2.00 (1.80–2.20)	<0.001
	Serum potassium [mmol/L]	4.20 (3.90–4.70)	4.20 (3.90–4.60)	4.20 (3.80–4.70)	4.30 (3.80–4.80)	4.20 (3.90–4.70)	<0.001
	Serum sodium [mmol/L]	139 (136–141)	139 (137–141)	139 (136–141)	139 (136–141)	139 (136–141)	<0.001
Observation	Heart Rate [BPM]	85.5 (75.0–98.0)	83.5 (75.0–91.0)	87.8 (74.0–103)	87.0 (74.0–104)	86.0 (74.0–99.0)	<0.001
	Left Atrial Dilation	4183 (44.5)	898 (30.3)	636 (17.9)	525 (94.3)	2124 (91.0)	<0.001
	Right Atrial Dilation	2813 (29.9)	371 (12.5)	230 (6.49)	439 (78.8)	1773 (75.9)	<0.001
Demographic	Age [years]	76.5 (67.3–83.6)	73.3 (65.1–80.5)	78.0 (68.7–85.1)	78.1 (69.2–85.4)	77.8 (68.9–84.3)	<0.001
	Male sex	5364 (57.1)	2136 (72.1)	1671 (47.1)	277 (49.7)	1280 (54.8)	<0.001
