# Supplementary material for: A Comparison of the Impact of Pharmacological Treatments on Cardioversion, Rate Control, and Mortality in Data-Driven Atrial Fibrillation Phenotypes in Critical Care
Source: Bioengineering (Basel). 2024 Feb 20;11(3):199. doi: 10.3390/bioengineering11030199 (PMC10967916; doi:10.3390/bioengineering11030199)
Supplement: Supplementary file 1 [file bioengineering-11-00199-s001.zip › bioengineering-2826818-supplementary.pdf]

1 Secondary Analysis

1.1 Methods

To identify possible differences between the clusters identified by hierarchical agglomerative clustering and KMeans, we perform a secondary analysis of the dataset using KMeans. We perform the clustering using *k* as determined in the primary analysis, re-run KMeans 100 times to identify the most common clustering solution and reject solutions where KMeans is stuck in local minima. The most common clustering solution is further analyzed.

1.2 Results

1.2.1 Patient Phenotypes

The cohort characteristics and cluster characteristics obtained from KMeans are presented in Table S1. The dominant characteristics of the patient clusters identified using KMeans are as follows.

Cluster 1 (n=806)
This cluster is characterized by the highest rate of renal insufficiencies and heart failure. In terms of laboratory measurements, patients in this cluster have the highest concentrations of creatinine, magnesium, and potassium.

Cluster 2 (n=3587)
Patients in this cluster are the oldest with a mean age of 78.6 years. They are primarily characterized by a particularly high rate of coronary artery atherosclerosis, hypertension, valvulopathies, myocardial infarction, and post-operative conditions. Nonetheless, patients in this cluster have the lowest rates of left and right atrial dilation, and the lowest heart rates.

Cluster 3 (n=2910)
Patients in this cluster are the youngest with an average age of 71 years and mostly male. They are characterized by a relatively low morbidity having the lowest rates of heart failure and thyroid disorders, and only slightly elevated rates of myocardial infarction.

Cluster 4 (n=2098)
Cluster 4 has the highest share of female patients as well as the highest rate of arrhythmia history. Patients in this cluster have have the highest rate of thyroid disorders, and the lowest rates of coronary artery atherosclerosis, myocardial infarction, and post-operative conditions.

Table S1. Characteristics of the cohort, and the identified clusters of the secondary analysis.

Category	Variable	Entire Cohort (n=9401)	Cluster 1 (n=806)	Cluster 2 (n=3587)	Cluster 3 (n=2910)	Cluster 4 (n=2098)	p-value (among clusters)
Medical History	Anaemia	1127 (11.99)	101 (12.5)	451 (12.6)	245 (8.42)	330 (15.7)	<0.001
	Arrhythmia History	1959 (20.8)	199 (24.7)	615 (17.2)	608 (20.8)	537 (25.6)	<0.001
	Collagen disease	115 (1.22)	13 (1.61)	44 (1.23)	17 (0.58)	41 (1.95)	<0.001
	COPD	1256 (13.4)	116 (14.4)	407 (11.3)	332 (11.4)	401 (19.1)	<0.001
	Cor pulmonale	754 (8.02)	77 (9.55)	298 (8.31)	198 (6.80)	181 (8.63)	<0.001
	Coronary artery atherosclerosis	3461 (36.8)	209 (25.9)	1789 (49.8)	1003 (34.4)	460 (21.9)	<0.001
	Diabetes	2147 (22.8)	218 (27.5)	748 (20.8)	636 (21.8)	545 (25.9)	<0.001
	Heart failure	4128 (43.9)	494 (61.3)	1412 (39.3)	1111 (38.1)	1111 (52.9)	<0.001
	Hypertension	4562 (48.5)	144 (17.9)	2072 (57.7)	1553 (53.3)	793 (37.8)	<0.001
	Myocardial infarction	933 (9.92)	94 (11.6)	379 (10.5)	290 (9.97)	170 (8.1)	<0.001
	OSA	403 (4.29)	41 (5.90)	112 (3.12)	155 (5.33)	95 (4.53)	<0.001
	Post-operative condition	3250 (34.6)	101 (12.5)	1932 (53.8)	981 (33.7)	236 (11.2)	<0.001
	Renal insufficiency	3261 (34.7)	734 (91.7)	810 (22.5)	697 (23.9)	1020 (48.6)	<0.001
	Respiratory failure	1450 (15.4)	190 (23.5)	332 (9.26)	425 (14.6)	503 (23.9)	<0.001
	Rheumatism	403 (4.29)	27 (3.35)	205 (5.72)	85 (2.92)	86 (4.10)	<0.001
	Sepsis	1144 (12.2)	215 (26.7)	184 (5.13)	280 (9.62)	465 (22.1)	<0.001
	Thyroid disorder	1051 (11.2)	101 (12.5)	405 (11.2)	260 (8.93)	285 (13.5)	<0.001
	Valvulopathy	2865 (30.5)	167 (20.7)	1496 (41.7)	740 (25.4)	462 (22.2)	<0.001
Laboratory Measurement	Erythrocyte count	3.88 (3.38-4.37)	3.70 (3.28-4.16)	3.71 (3.32-4.00)	4.57 (4.28-4.88)	3.35 (2.97-3.70)	<0.001
	Erythrocyte distribution width	14.4 (13.5-15.7)	16.0 (14.6-17.6)	14.0 (13.3-14.7)	13.8 (13.3-14.6)	16.3 (15.0-17.8)	<0.001
	Haemoglobin	12.1 (10.6-13.4)	11.1 (9.8-12.4)	11.9 (11.1-12.7)	13.9 (13.1-14.8)	10.0 (8.90-10.8)	<0.001
	Leukocyte count	10.0 (7.30-13.7)	10.5 (7.4-15.4)	9.20 (6.80-12.3)	10.5 (7.80-14.2)	10.8 (7.40-15.14)	<0.001
	Platelet count	215 (163-279)	218 (156-296)	185 (144-229)	233 (189-289)	261 (182-355)	<0.001
	Prothrombin time	14.5 (13.1-18.1)	17.2 (13.6-29.1)	14.4 (13.1-16.6)	13.9 (12.8-17.1)	15.1 (13.4-20.8)	<0.001
	Serum calcium	8.60 (8.10-9.10)	8.70 (8.20-9.30)	8.50 (8.10-8.90)	8.80 (8.40-9.30)	8.40 (7.90-8.80)	<0.001
	Serum creatinine	1.10 (0.80-1.50)	3.60 (2.40-5.50)	1.00 (0.80-1.30)	1.00 (0.80-1.30)	1.20 (0.90-1.70)	<0.001
	Serum magnesium	2.00 (1.80-2.20)	2.20 (1.90-2.50)	2.00 (1.80-2.20)	1.90 (1.80-2.10)	1.90 (1.70-2.20)	<0.001
	Serum potassium	4.20 (3.90-4.70)	5.20 (4.50-6.10)	4.20 (3.80-4.50)	4.20 (3.80-4.60)	4.20 (3.80-4.64)	<0.001
	Serum sodium	139 (136-141)	136 (133-139)	139 (137-141)	139 (137-141)	138 (135-141)	<0.001
Observation	Heart Rate	85.5 (75.0-98.0)	83.0 (71.1-98.9)	79.9 (71.0-87.9)	88.5 (78.0-104)	92.9 (79.9-109)	<0.001
	Left Atrial Dilation	4183 (44.5)	433 (53.7)	1508 (42.0)	1296 (44.5)	946 (45.0)	<0.001
	Right Atrial Dilation	2813 (29.9)	324 (40.2)	920 (25.6)	905 (31.1)	664 (31.6)	<0.001
Demography	Age	76.5 (67.3-83.6)	76.6 (68.1-84.0)	78.6 (71.5-84.6)	71.0 (61.7-79.8)	78.2 (69.2-84.8)	<0.001
	Male sex	5364 (57.1)	499 (61.9)	1992 (55.5)	1903 (65.4)	970 (46.2)	<0.001

1.2.2 Average Treatment Effects

Rhythm Control

Rhythm control was best achieved using PCBs (9.78%/d [8.74-11.0]), followed by CCBs (4.73%/d [4.04-5.74]), BBs and MgS (2.28%/d [1.81-2.61], 1.90%/d [1.44-2.54]).

The efficacy of PCBs varied among clusters, with cluster 2 showing the highest conversion rates (11.2%/d [9.50-13.5]). BBs and MgS universally showed the lowest conversion rates below 4%/h. Even though ATEs differed among the identified clusters, the general order of treatment efficacy was preserved in each cluster.

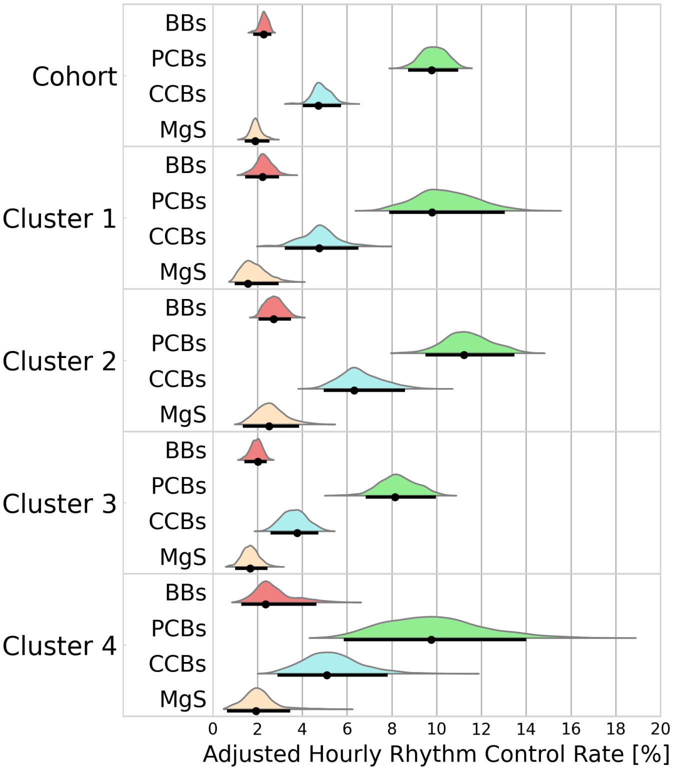

Figure S1. Probability distributions of adjusted hourly rhythm control rates of the secondary analysis. Black dots represent the distribution modes. Black lines represent the 95% highest density intervals. BBs - beta blockers; PCBs - potassium channel blockers; CCBs - calcium channel blockers; MgS - magnesium sulphate.

Rate Control Ventricular rate was best controlled using CCBs when considering the complete cohort (18.8%/h [16.4-22.8]), followed by MgS (15.6%/h [11.4-19.2]) and PCBs (15.2%/h [13.2-17.5]. The poorest effect was observed with BBs (12.7%/h [11.2-14.6]).

The efficacy of AADs varied across clusters. While CCBs maintained superior efficacy in 3 clusters, PCBs and MgS showed similar or better rate control in cluster 4 (13.1%/h (9.23-25.0), 14.5%/h (4.76-32.6) vs. 13.3%/h [5.93-20.4]).

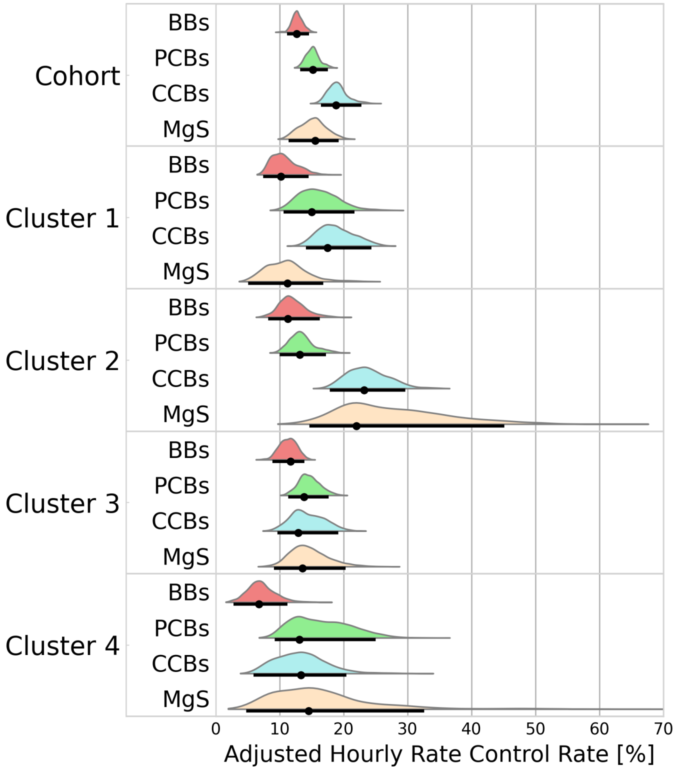

Figure S2. Probability distributions of adjusted hourly rate control rates of the secondary analysis. Black dots represent the distribution modes. Black lines represent the 95% highest density intervals. BBs - beta blockers; PCBs - potassium channel blockers; CCBs - calcium channel blockers; MgS - magnesium sulphate.

Mortality Mortality rates were the lowest in patients receiving PCBs (0.95%/d (0.78-1.18)), followed by BBs, CCBs and MgS (1.28%/d (1.00-1.51), 1.40%/d (1.04-1.88), 1.45%/d (1.07-1.99)). While the association of PCB wit the lowest mortality can be observed in the majority of clusters, BBs show a tendency towards lower mortality in cluster 4 (1.07%/d [0.56-1.90], 2.09%/d [0.77-3.05]).

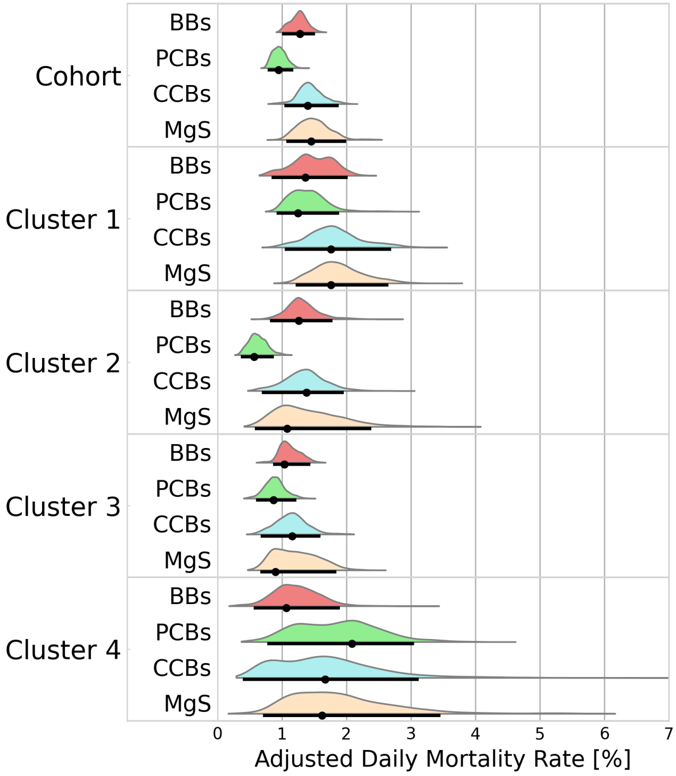

Figure S3. Probability distributions of adjusted daily mortality rates of the secondary analysis. Black dots represent the distribution modes. Black lines represent the 95% highest density intervals. BBs - beta blockers; PCBs - potassium channel blockers; CCBs - calcium channel blockers; MgS - magnesium sulphate.

1.3 Discussion

Similarly to the analysis using hierarchical agglomerative clustering, KMeans revealed recognizable phenotypes. For example, cluster 4, which was associated with the highest rate of thyroid disorders, had the highest share of female patients. This is in line with previous studies suggesting that thyroid disorders are more common in women than in men. Similarly, cluster 2 was associated with the highest rate of coronary artery atherosclerosis, myocardial infarction, and postoperative conditions. The interaction of atherosclerosis and myocardial infarction is well established, and patients with myocardial infarction will commonly receive stents.

The identified clusters differed from the clusters captured in the primary analysis, This may be attributed to the different nature of the algorithms, as well as different distance metrics used. While the primary analysis used Gower's distance to accommodate continuous and categorical variables, KMeans uses the euclidean distance between observations.

Table S2. Covariate means before and after inverse probability treatment weighting for rhythm control and mortality.

Category	Variable	Unweighted Means				Weighted Means				Maximum absolute pairwise SMD unweighted	Maximum absolute pairwise SMD weighted
		BBs	PCBs	CCBs	MgS	BBs	PCBs	CCBs	MgS		
Medical History	Anaemia	0.13	0.13	0.11	0.12	0.13	0.12	0.12	0.11	0.05	0.06
	Arrhythmia History	0.30	0.14	0.24	0.30	0.25	0.20	0.23	0.28	0.22	0.11
	Collagen disease	0.01	0.02	0.02	0.01	0.01	0.02	0.01	0.01	0.05	0.03
	COPD	0.14	0.12	0.16	0.14	0.14	0.13	0.15	0.13	0.06	0.05
	Cor pulmonale	0.11	0.09	0.07	0.10	0.10	0.09	0.09	0.12	0.05	0.07
	Coronary artery atherosclerosis	0.29	0.52	0.18	0.23	0.32	0.38	0.30	0.30	0.39	0.12
	Diabetes	0.25	0.21	0.22	0.23	0.25	0.21	0.23	0.21	0.06	0.07
	Heart failure	0.54	0.42	0.42	0.54	0.50	0.46	0.45	0.51	0.13	0.06
	Hypertension	0.48	0.51	0.45	0.42	0.49	0.46	0.48	0.42	0.12	0.10
	Myocardial infarction	0.08	0.16	0.08	0.08	0.09	0.13	0.11	0.11	0.16	0.08
	OSA	0.05	0.03	0.05	0.05	0.05	0.03	0.05	0.05	0.06	0.06
	Post-operative condition	0.21	0.64	0.08	0.11	0.29	0.36	0.25	0.24	0.68	0.20
	Renal insufficiency	0.41	0.37	0.39	0.41	0.39	0.43	0.38	0.38	0.06	0.06
	Respiratory failure	0.21	0.15	0.27	0.23	0.20	0.21	0.24	0.21	0.17	0.07
	Rheumatism	0.06	0.05	0.02	0.04	0.05	0.05	0.03	0.05	0.10	0.09
	Sepsis	0.14	0.13	0.19	0.23	0.14	0.18	0.16	0.19	0.17	0.08
	Thyroid disorder	0.13	0.08	0.13	0.11	0.11	0.10	0.13	0.10	0.08	0.06
	Valvulopathy	0.31	0.47	0.17	0.24	0.32	0.38	0.27	0.28	0.32	0.12
Laboratory Measurement	Erythrocyte count	3.96	3.88	3.94	3.84	3.93	3.90	3.93	3.94	0.07	0.05
	Erythrocyte distribution width	15.2	14.6	15.1	15.2	15.0	14.9	14.9	15.0	0.21	0.06
	Haemoglobin	12.0	12.3	11.9	11.6	12.0	12.1	12.0	12.1	0.16	0.04
	Leukocyte count	11.9	11.3	13.4	12.5	12.1	11.7	12.2	12.0	0.11	0.04
	Platelet count	237	222	254	239	233	234	241	236	0.18	0.04
	Prothrombin time	20.5	15.7	17.9	21.8	18.8	17.2	18.1	18.6	0.29	0.11
	Serum calcium	8.66	8.56	8.51	8.52	8.60	8.55	8.52	8.56	0.09	0.07
	Serum creatinine	1.45	1.39	1.45	1.50	1.43	1.48	1.40	1.42	0.06	0.04
	Serum magnesium	2.02	2.09	1.94	1.86	2.00	2.03	1.96	1.94	0.26	0.13
	Serum potassium	4.35	4.29	4.36	4.39	4.32	4.32	4.33	4.32	0.11	0.03
	Serum sodium	138	139	138	138	138	139	138	138	0.10	0.03
Observation	Heart Rate	93.0	89.9	101	90.9	93.1	92.9	94.8	93.1	0.34	0.07
	Left Atrial Dilation	0.55	0.52	0.50	0.55	0.53	0.54	0.51	0.56	0.05	0.08
	Right Atrial Dilation	0.44	0.31	0.34	0.45	0.41	0.35	0.35	0.45	0.16	0.16
Demographic	Age	77.6	73.2	73.9	77.4	75.7	74.5	74.8	76.2	0.21	0.10
	Male sex	0.55	0.60	0.48	0.58	0.58	0.56	0.53	0.60	0.17	0.09

Table S3. Covariate means before and after inverse probability treatment weighting for rate control.

Category	Variable	Unweighted Means				Weighted Means				Maximum absolute pairwise SMD unweighted	Maximum absolute pairwise SMD weighted
		BBs	PCBs	CCBs	MgS	BBs	PCBs	CCBs	MgS		
Medical History	Anaemia	0.11	0.12	0.12	0.12	0.13	0.14	0.13	0.11	0.07	0.06
	Arrhythmia History	0.28	0.26	0.23	0.24	0.13	0.25	0.17	0.26	0.19	0.12
	Collagen disease	0.02	0.01	0.02	0.01	0.01	0.01	0.01	0.01	0.07	0.04
	COPD	0.14	0.16	0.13	0.16	0.13	0.15	0.14	0.15	0.07	0.07
	Cor pulmonale	0.09	0.07	0.07	0.09	0.09	0.09	0.09	0.10	0.10	0.11
	Coronary artery atherosclerosis	0.27	0.16	0.30	0.29	0.52	0.23	0.37	0.32	0.39	0.13
	Diabetes	0.23	0.23	0.23	0.24	0.21	0.24	0.22	0.25	0.08	0.11
	Heart failure	0.57	0.41	0.48	0.43	0.41	0.55	0.44	0.42	0.21	0.09
	Hypertension	0.46	0.46	0.47	0.50	0.50	0.39	0.46	0.48	0.19	0.06
	Myocardial infarction	0.08	0.08	0.10	0.11	0.16	0.11	0.14	0.17	0.14	0.19
	OSA	0.05	0.05	0.04	0.05	0.03	0.01	0.03	0.01	0.19	0.17
	Post-operative condition	0.17	0.09	0.28	0.25	0.63	0.15	0.38	0.29	0.61	0.20
	Renal insufficiency	0.43	0.41	0.39	0.39	0.39	0.52	0.44	0.39	0.20	0.08
	Respiratory failure	0.25	0.30	0.22	0.26	0.16	0.32	0.22	0.25	0.20	0.09
	Rheumatism	0.06	0.03	0.05	0.02	0.04	0.06	0.04	0.05	0.09	0.08
	Sepsis	0.17	0.20	0.16	0.18	0.14	0.29	0.20	0.20	0.31	0.16
	Thyroid disorder	0.12	0.12	0.11	0.11	0.08	0.11	0.10	0.11	0.10	0.08
	Valvulopathy	0.30	0.16	0.32	0.25	0.44	0.22	0.36	0.25	0.33	0.16
Laboratory Measurement	Erythrocyte count	3.94	3.94	3.91	3.94	3.87	3.81	3.89	3.99	0.13	0.11
	Erythrocyte distribution width	15.2	15.0	15.0	14.9	14.6	15.4	14.9	14.8	0.26	0.05
	Haemoglobin	13.0	12.0	12.0	12.0	12.2	11.5	12.1	12.2	0.33	0.06
	Leukocyte count	11.6	13.9	11.9	12.5	11.2	12.0	11.9	11.9	0.18	0.05
	Platelet count	236	251	235	242	226	237	234	231	0.14	0.05
	Prothrombin time	20.3	17.8	18.1	17.5	15.7	20.0	16.6	17.6	0.24	0.12
	Serum calcium	8.62	8.50	8.56	8.50	8.55	8.45	8.52	8.54	0.18	0.07
	Serum creatinine	1.49	1.45	1.44	1.39	1.41	1.56	1.47	1.41	0.12	0.04
	Serum magnesium	2.01	1.95	1.99	1.97	2.08	1.86	2.03	1.92	0.23	0.15
	Serum potassium	4.33	4.38	4.33	4.33	4.30	4.38	4.31	4.32	0.07	0.05
	Serum sodium	139	138	138	138	139	138	138	138	0.11	0.04
Observation	Heart Rate	98.7	101	96.3	97.8	90.0	101	94.8	97.4	0.26	0.09
	Left Atrial Dilation	0.58	0.53	0.56	0.54	0.51	0.62	0.54	0.56	0.20	0.06
	Right Atrial Dilation	0.45	0.38	0.40	0.37	0.31	0.52	0.35	0.46	0.28	0.20
Demographic	Age	77.1	74.0	75.3	74.1	72.9	76.0	73.9	74.8	0.24	0.08
	Male sex	0.53	0.49	0.55	0.51	0.60	0.52	0.57	0.57	0.13	0.07

Table S4. Average treatment effects for the entire cohort and phenotypes - rhythm control.

		**Unadjusted hourly rhythm conversion rate**	**Adjusted hourly rhythm conversion rate**
Cohort	BBs	2.30 (1.95-2.51)	2.28 (1.81-2.61)
	PCBs	9.75 (8.85-10.9)	9.78 (8.74-11.0)
	CCBs	4.85 (4.28-5.47)	4.73 (4.04-5.74)
	MgS	1.90 (1.46-2.32)	1.90 (1.44-2.54)
Cluster 1	BBs	3.05 (2.37-3.62)	2.93 (2.12-3.88)
	PCBs	11.5 (9.85-13.2)	11.6 (9.35-13.3)
	CCBs	5.97 (3.37-7.78)	5.33 (2.94-8.32)
	MgS	2.28 (0.62-3.72)	2.67 (0.36-3.88)
Cluster 2	BBs	2.34 (1.89-2.81)	2.21 (1.77-2.97)
	PCBs	10.5 (8.24-13.0)	9.73 (8.37-13.5)
	CCBs	5.34 (4.18-6.48)	5.22 (3.62-6.58)
	MgS	1.70 (1.25-2.40)	1.69 (1.17-2.68)
Cluster 3	BBs	2.42 (1.37-3.26)	2.27 (1.33-3.63)
	PCBs	8.29 (5.79-13.7)	7.39 (5.16-14.7)
	CCBs	3.28 (1.38-5.76)	3.50 (1.17-6.25)
	MgS	2.05 (1.18-3.73)	1.98 (0.98-4.22)
Cluster 4	BBs	1.84 (1.36-2.23)	1.90 (1.15-2.35)
	PCBs	7.62 (6.20-8.74)	7.69 (5.80-9.22)
	CCBs	4.46 (3.59-5.62)	4.38 (3.48-6.02)
	MgS	1.87 (1.34-2.42)	1.74 (1.20-2.67)

Table S5. Average treatment effects for the entire cohort and phenotypes - rate control.

		**Unadjusted hourly rate control rate**	**Adjusted hourly rate control rate**
Cohort	BBs	12.2 (11.4-14.0)	12.7 (11.2-14.6)
	PCBs	14.6 (13.8-16.5)	15.2 (13.2-17.5)
	CCBs	19.1 (16.8-21.2)	18.8 (16.4-22.8)
	MgS	15.0 (12.1-18.0)	15.6 (11.4-19.2)
Cluster 1	BBs	11.9 (9.30-15.6)	11.5 (8.70-16.3)
	PCBs	15.2 (11.4-16.6)	12.9 (11.1-17.8)
	CCBs	20.1 (15.3-30.7)	19.4 (14.4-31.8)
	MgS	15.6 (10.4-27.9)	15.0 (9.76-30.8)
Cluster 2	BBs	12.1 (10.2-14.9)	12.6 (9.62-15.3)
	PCBs	12.6 (9.99-14.9)	12.4 (9.15-16.8)
	CCBs	20.5 (16.7-23.3)	20.4 (15.9-25.1)
	MgS	15.7 (10.9-23.1)	17.6 (9.11-25.4)
Cluster 3	BBs	13.4 (6.92-17.3)	9.66 (5.54-18.7)
	PCBs	13.4 (8.80-23.6)	13.5 (7.66-27.6)
	CCBs	18.1 (12.1-27.9)	18.3 (11.2-32.6)
	MgS	19.7 (8.51-61.0)	19.9 (7.92-57.5)
Cluster 4	BBs	13.8 (10.7-15.7)	13.9 (10.8-17.0)
	PCBs	18.6 (16.0-22.4)	18.2 (14.9-23.6)
	CCBs	16.7 (14.4-19.6)	16.7 (12.6-20.9)
	MgS	13.4 (8.86-16.1)	11.2 (8.40-17.8)

Table S6. Average treatment effects for the entire cohort and phenotypes - in-hospital mortality.

		**Unadjusted daily mortality rate**	**Adjusted hourly mortality rate**
Cohort	BBs	1.27 (1.03-1.43)	1.28 (1.00-1.51)
	PCBs	0.96 (0.80-1.15)	0.95 (0.78-1.18)
	CCBs	1.40 (1.10-1.72)	1.40 (1.04-1.88)
	MgS	1.50 (1.17-1.88)	1.45 (1.07-1.99)
Cluster 1	BBs	0.80 (0.46-1.16)	0.81 (0.37-1.39)
	PCBs	0.53 (0.30-0.70)	0.52 (0.28-0.76)
	CCBs	0.56 (0.08-0.94)	0.44 (0.05-1.15)
	MgS	1.03 (0.45-1.88)	0.80 (0.33-1.95)
Cluster 2	BBs	1.46 (1.16-1.83)	1.33 (1.04-1.93)
	PCBs	1.72 (1.23-2.17)	1.68 (1.10-2.24)
	CCBs	1.78 (1.51-2.37)	2.03 (1.41-2.51)
	MgS	1.76 (1.31-2.34)	1.76 (1.18-2.65)
Cluster 3	BBs	1.61 (1.12-2.46)	1.50 (0.95-2.62)
	PCBs	1.44 (0.81-2.14)	1.39 (0.68-2.44)
	CCBs	2.20 (1.02-3.15)	2.12 (0.79-3.26)
	MgS	1.99 (0.69-3.66)	1.99 (0.59-3.87)
Cluster 4	BBs	1.10 (0.79-1.43)	1.13 (0.82-1.55)
	PCBs	0.84 (0.59-1.03)	0.78 (0.54-1.13)
	CCBs	0.93 (0.59-1.38)	1.02 (0.45-1.37)
	MgS	1.02 (0.70-1.70)	0.93 (0.60-1.80)
